# The *MCP‐1* rs1024611 and *MTHFR* rs1801133 gene variations and expressions in alopecia areata: A pilot study

**DOI:** 10.1002/iid3.564

**Published:** 2021-11-09

**Authors:** Pardis‐Sadat Tabatabaei‐Panah, Hamideh Moravvej, Mahsa Hajihasani, Mahsa Mousavi, Ralf J. Ludwig, Reza Akbarzadeh

**Affiliations:** ^1^ Department of Biology, East Tehran Branch Islamic Azad University Tehran Iran; ^2^ Skin Research Center Shahid Beheshti University of Medical Sciences Tehran Iran; ^3^ Lübeck Institute of Experimental Dermatology University of Lübeck Germany; ^4^ Institute of Clinical Chemistry and Laboratory Medicine University Medical Center Hamburg‐Eppendorf Hamburg Germany

**Keywords:** alopecia areata, gene expression, MCP‐1, MTHFR, polymorphism

## Abstract

**Background:**

Monocyte chemoattractant protein‐1 (MCP‐1) is highly expressed by lymphocytes at skin sites affected by alopecia areata (AA). Variations in *MCP‐1* as well as in methylene‐tetrahydrofolate reductase (*MTHFR*), a key enzyme related to many inflammatory pathologies, have been associated with several autoimmune disorders. This study was designed to test a possible association between *MCP‐1* and *MTHFR* variations and altered expression of their genes and the risk of AA.

**Methods:**

Blood samples of patients (60) suffering from AA as well as healthy subjects (60) were collected. Gene expression levels of *MCP‐1* and *MTHFR* were evaluated by real‐time reverse‐transcription polymerase chain reaction analysis. Moreover, *MCP‐1* rs1024611 (A‐2518G) and *MTHFR* rs1801133 (C677T) polymorphisms were genotyped by using polymerase chain reaction‐restriction fragment length polymorphism assays.

**Results:**

In contrast to *MCP‐1*, the *MTHFR* gene expression was found to be significantly higher in patients than in controls. Further stratification of the patients revealed that polymorphic genotypes in *MCP‐1* (AG + GG) and *MTHFR* (CT + TT) could significantly alter gene expression levels. Elevation of *MCP‐1* expression was significantly associated with the total number of variant *MCP‐1* and *MTHFR* alleles. However, no statistically significant difference was noticed in the genotypic distribution of *MCP‐1* and *MTHFR* variations between patients and controls.

**Conclusion:**

In summary, despite *MCP‐1* rs1024611 and *MTHFR* rs1801133 variations are not associated with AA risk, they may implicate the disease pathogenesis by influencing *MCP‐1* activity.

## Graphical abstract

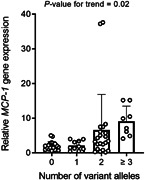

## 1 INTRODUCTION

Alopecia areata (AA) is a common cause of nonscarring hair loss. AA is a chronic inflammatory, T cell‐mediated autoimmune disease, affecting both the hair follicles and the nail apparatus.^1^, ^2^ It affects approximately 2% of the general people, occurring in both males and females.^3^ The onset of AA may happen at any age, but the majority of patients are under the age of 40 years.^3^ Although the genome‐wide association studies (GWAS) have identified associations of several genes with AA,^4^, ^5^, ^6^ a few of them have been so far reported as a cause of the disease or functionally validated for the pathogenesis of AA.^7^

In AA, lymphocyte infiltration contributes to changes in the hair follicles, which may cause hair loss.^8^ Herein, altered expressions of chemokines in the hair bulbs are important in attracting immune cells around the hair bulb.^9^ It has been shown that the expression of monocyte chemoattractant protein‐1 (MCP‐1) is increased in AA.^1^ The MCP‐1, a chemokine encoded by the *C‐C Motif Chemokine Ligand 2* (*CCL2*) gene, is released by lymphocytes and monocytes at sites of injury and inflammation, which is important for the recruitment of leukocytes, especially monocytes and T cells in acute inflammation and as a mediator in chronic inflammatory conditions.^10^

Given the importance of MCP‐1 chemokine in AA, in vitro studies have connected the aberrant expression levels of MCP‐1 chemokine and concentrations of homocysteine and folate.^11^, ^12^, ^13^ Methylenetetrahydrofolate reductase (MTHFR) is a crucial enzyme in folate metabolism and deficiency in folate metabolism could increase the risk of AA development.^14^, ^15^ The *MTHFR* gene influences the process of nucleic acid synthesis as well as DNA methylation, which is associated with autoimmune disorders.^16^ It has been shown that the folate concentrations of red blood cells are significantly lower in AA patients compared to healthy individuals.^17^ Moreover, lower concentrations were found in patients with alopecia totalis or universalis (that are more severe forms of the disease) compared to AA patients with patchy hair loss.^17^

It is believed that genetic variations in the regulatory regions of the *MCP‐1* and *MTHFR* genes could affect the susceptibility to autoimmune diseases. More specifically, two common polymorphisms in *MCP‐1* (rs1024611) and *MTHFR* (rs1801133) have been reported to be involved in various autoimmune diseases.^18^, ^19^ Nevertheless, the genetic contribution of *MCP‐1* and *MTHFR* genes has not been investigated in AA in detail. So far, two studies analyzed either the *MCP‐1* (rs1024611)^20^ or *MTHFR* (rs1801133)^21^ polymorphisms in AA and found no association between AA and the *MCP‐1* variation. To our best of knowledge, these polymorphisms have never been studied in AA in an Iranian population. Therefore, this study was designed to uncover a possible association between *MCP‐1* (rs1024611) and *MTHFR* (rs1801133) variations and susceptibility to AA disease in an Iranian cohort. Furthermore, the influence of these variations in altered levels of *MCP‐1* and *MTHFR* gene expressions was investigated.

## 2 MATERIALS AND METHODS

### 2.1 Subjects

Human Research Ethics Committee of Skin Research Center, Shahid Beheshti University of Medical Sciences approved this study and conformed to the ethical guidelines of the 1975 Declaration of Helsinki. Sixty blood samples were collected from patients suffering from AA in Shohada Tajrish and Loghman Hakim hospitals in Tehran. This cohort has been previously described.^22^, ^23^ AA diagnosis was made by a board‐certified dermatologist based on the history and clinical presentation, dermoscopy, and histopathological (if required) evaluations according to AA investigational assessment guidelines.^24^ Samples were taken immediately after diagnosis of the AA. Patients who were included in the study did not take any treatment regarding AA and subjects who received steroids, immunosuppressive drugs, or blood transfusion as well as individuals who suffered from other skin or autoimmune disorders were excluded from the study. Similarly, patients with hair loss due to other reasons rather than AA were also excluded. A total of 60 healthy subjects, who had no clinical evidence of AA, were recruited as controls. All patients and controls gave written informed consent before entering the study. Information including age, gender, nail dystrophy, focal infection, eczema, family history, and anemia was obtained from patients with AA and controls. All subjects with inappropriate processing of laboratory analysis or lacking demographic data were excluded from the study. Blood samples (3 ml) of the subjects were collected in a tube containing ethylene diamine tetra‐acetic acid (EDTA) and stored at −20°C until further use.

### 2.2 Genetic evaluation of *MCP‐1* and *MTHFR* genotyping

Polymorphisms of *MCP‐1* (rs1024611) and *MTHFR* (rs1801133) were carried out by the polymerase chain reaction‐restriction fragment length polymorphism (PCR‐RFLP) method of a genomic DNA fragment in AA patients and control subjects. Genomic DNA was extracted from peripheral blood samples using the DNA blood extraction reagent kit according to the kit procedure (DNGTM – Plus). The primer sequences as well as PCR procedure for *MCP‐1* and *MTHFR* were obtained from the previous reports,^20^, ^21^ with some modification. Briefly, PCR was performed using master mix containing reaction buffer, 4 mM MgCl2, 0.4 mM dNTP, and 0.05 U/µl Taq DNA‐polymerase (Fermentas), 0.4 mM of primers (Bioneer), 10 µg of DNA template, and distilled water up to a final volume of 25 µl. The following thermal profiles were applied for *MCP‐1*: 94°C for 1 min, 55°C for 35 s, and 72°C for 45 s, and for *MTHFR*: 94°C for 30 s, 61°C for 30 s, and 72°C for 30 s. Following a final extension of 5 min at 72°C, the PCR products were run on a 3% agarose gel and stained with SYBR Green (CinnaGen). PCR products of *MCP‐1* and *MTHFR* were respectively digested with 10 U of *Pvu*II and *Hinf*I (Thermo Fischer Scientific) in 10× buffer and H_2_O up to a final volume of 30 µl at 37°C for 16 h. Following digestion, individuals with wild‐type genotype (CC), homozygote variant (TT), and heterozygote variant (CT) of *MCP‐1* provided respectively a fragment of 198 bp, two fragments of 175/23 bp, and three fragments of 198/175/23 bp. For *MTHFR*, subjects indicating a single fragment of 930 bp, two fragments of 708/222 bp, and three fragments of 930/708/222 were respectively identified as wild‐type genotype (AA), homozygote variant (GG), and heterozygote variant (AG).

### 2.3 Expression analysis of *MCP‐1* and *MTHFR* genes

Total RNA from patient and control subjects was extracted by the RNX‐Plus kit (SinaClon, Iran) according to the manufacturer's instructions. Briefly, 1 ml ice‐cold RNX^tm^‐Plus was added to the blood samples and homogenized. After incubation for 5 min, 200 µl chloroform (Merck) was added and incubated for 5 min at 4°C. Samples were centrifuged at 12,000 rpm at 4°C for 15 min. The aqueous phase was transferred to an RNase‐free tube and an equal volume of isopropanol (Merck) was added. After 15 min incubation, samples were centrifuged at 12,000 rpm at 4°C for 15 min. The pellet was dissolved in 50 µl sterile water. The concentration of the RNA was determined by using a spectrometer (BioSpectrometer, Eppendorf). The cDNA synthesis was performed by using 10x Molony Murine Leukemia Virus (M‐MuLV) buffer (CinnaGen, Iran), 200 U/µl M‐MuLV enzyme (CinnaGen), 0.2 µg Random Hexamer Primer (Bioneer), 0.5 µg Oligo dT (CinnaGen), 0.5 mM deoxy‐nucleotide triphosphate (dNTP) (CinnaGen), 2 µg RNA, and nuclease‐free water were added in two steps into a sterile, nuclease‐free tube on ice. Tubes were mixed gently, centrifuged briefly, and incubated at 42°C for one hour. Tubes were then incubated for 10 min at 80°C to inactivate the enzyme.

Gene expression analysis of *MCP‐1* and *MTHFR* was performed by real‐time PCR according to the guidelines previously reported,^25^ by using SYBR Green Master Mix (SinaClon) assay. The housekeeping glyceraldehyde‐3‐phosphate dehydrogenase (*GAPDH*) gene served as control. Primer sequences (forward and reverse) were designed by ABI PCR equipment: 5′‐TCA AAC TGA AGC TCG CAC TC‐3′ and 5′‐ATT GAT TGC ATC TGG CTG AG‐3′ for *MCP‐1*, 5′‐ GCT TCC GAG AGT GGA GAA AC‐3′ and 5′‐GTG CTT CTT CCC AAG GAG AG‐3′ for *MTHFR*, and 5′‐GAA GGT GAA GGT CGG AGT‐3′ and 5′‐GAA GAT GGT GAT GGG ATT TC‐3 for *GAPDH* (as housekeeping gene). For the PCR reaction, a final volume of 20 µl containing 10 µl of SYBR Green PCR Master Mix (Fermentas), 0.4 mM of primers, 1 µl of cDNA, and distilled water was prepared. After an initial 10 min denaturation at 95°C, the cDNA products of *MCP‐1* and *MTHFR* genes were respectively amplified with 35 and 25 cycles of denaturation at 95°C for 15 s, and annealing and extension at 60°C for 1 min. The specificity of the PCR was controlled by melt curve analysis and adequate negative controls. Gene expression levels of samples were normalized to *GAPDH* expression as the reference gene according to the relative quantification method 2^−∆∆Ct^ and fold change values were obtained.

### 2.4 Statistical analysis

Statistical analysis was performed using SPSS (IBM SPSS Statistics, IBM Corporation) and GraphPad Prism (GraphPad Software, Inc.) software. Results were given as the mean ± *SD* or *SE*. The mean quantity values in patients and controls were compared by independent sample *t*‐test. The *χ* ^2^ test was used to analyze the possible deviation from the Hardy–Weinberg equilibrium. Genetic distribution of the genotypes between patients and controls as well as possible association between clinical‐demographic parameters and polymorphic genotypes in the patients were analyzed by logistic regression analysis and odds ratio (OR) and 95% confidence interval (CI) were calculated. To analyze the effect of polymorphic alleles of *MCP‐1* rs1024611 or *MTHFR* rs1801133 variations on *MCP‐1* expression, analysis of variance test was applied for four subgroups of individuals with 0 (AA + CC), 1 (AG + CC, AA + CT), 2 (AA + TT, AG + CT, GG + CC), ≥3 (AG + TT, GG + CT, and GG + TT) variants, where genotypes are respectively presented for *MCP‐1* and *MTHFR*. A *p*‐value of less than 0.05 was considered statistically significant.

## 3 RESULTS

### 3.1 Study power and Hardy–Weinberg equilibrium analysis

This cohort study included 60 patients suffering from AA (mean age ± *SE* = 28.4 ± 1.4) and 60 healthy subjects (mean age ± *SE* = 30.1 ± 0.7). Based upon the reported prevalence of the variants and considering the medium effect sizes of 0.35 (http://www.ncbi.nlm.nih.gov/snp), the sample size of our study has a power of 85% at a significance level of 0.05. Hardy–Weinberg equilibrium analysis indicated that the distribution of *MCP‐1* (*p* = .08) and *MTHFR* (*p* = .45) genotypes were consistent with the equilibrium.

### 3.2 *MTHFR* gene expression is associated with AA

**Figure 1:**
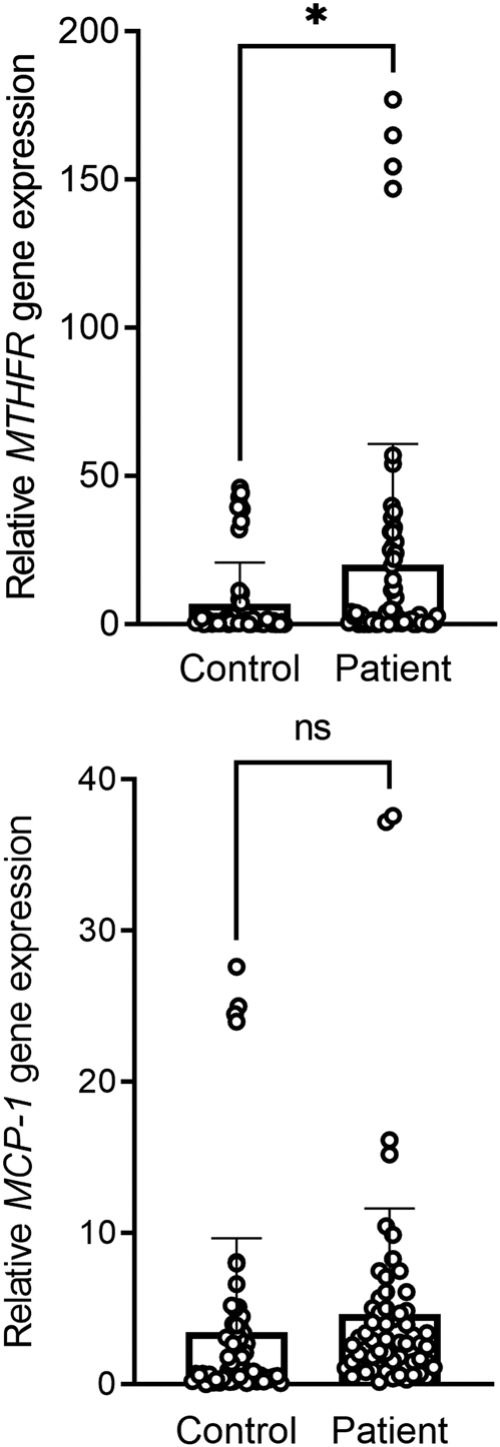
Relative *MCP‐1* and *MTHFR* gene expression in AA patients and control subjects. Expression levels are presented as mean ± *SD*. Asterisk indicate a significant *p*‐value (*p* < .05). AA, alopecia areata; MCP‐1, monocyte chemoattractant protein‐1; MTHFR, methylene‐tetrahydrofolate reductase; ns, nonsignificant; *SD*, standard deviation

Quantitative gene expression analysis was performed to scrutinize the influence of *MCP‐1* and *MTHFR* expressions in AA (Figure 1). A significant upregulation was found in the expression level of the *MTHFR* gene between the patient and control groups (*p* = .02). In contrast, the difference in *MCP‐1* gene expression levels was not statistically significant between patients and healthy individuals (*p* = .31). These data indicate that *MTHFR* gene could be involved in the pathogenesis of AA.

### 3.3 Polymorphic genotypes are associated with increased *MCP‐1* and *MTHFR* gene expression in AA

**Figure 2:**
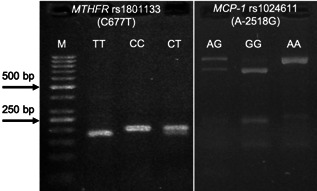
Sample agarose gel electrophoresis for detection of *MCP‐1* rs1024611 (A‐2518G) and *MTHFR* rs1801133 (C677T) variations by PCR‐RFLP, digested with *Pvu*II and *Hinf*I, respectively. M: 100 bp marker. CC: wild‐type genotype (198 bp), TT: homozygote variant (175/23 bp), and CT: heterozygote variant (198/175/23 bp) for *MTHFR* polymorphism. AA: wild‐type genotype (930 bp), GG: homozygote variant (708/222 bp), and AG: heterozygote variant (930/708/222) for *MCP‐1* variation. MCP‐1, monocyte chemoattractant protein‐1; MTHFR, methylene‐tetrahydrofolate reductase; PCR‐RFLP, polymerase chain reaction‐restriction fragment length polymorphism

**Figure 3:**
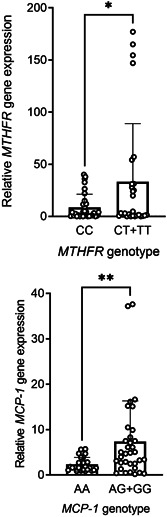
Relative *MCP‐1* rs1024611 and *MTHFR* rs1801133 gene expression between wild and polymorphic genotypes in the patient group. Expression levels are presented as mean ± *SD*. Asterisk indicates a significant *p*‐value (*p* < .05). MCP‐1, monocyte chemoattractant protein‐1; MTHFR, methylene‐tetrahydrofolate reductase; *SD*, standard deviation

Since the variations in the genes could potentially affect the levels of expression, PCR‐RFLP method was applied to analyze the variations of *MCP‐1* (rs1024611) and *MTHFR* (rs1801133). A sample gel is shown in Figure 2. To analyze the effect of minor‐allele in both genes, patients were subdivided into groups with wild‐ or polymorphic genotypes. Of note, patients with polymorphic genotypes in *MCP‐1* (AG + GG) and *MTHFR* (CT + TT) had a higher gene expression than those with wild genotypes (AA and CC in *MCP‐1* and *MTHFR*, respectively), which was statistically significant (*p* = .006 for *MCP‐1* and *p* = .01 for *MTHFR*, Figure 3).

### 3.4 Polymorphic genotypes of *MCP‐1* and *MTHFR* are associated with the altering levels of *MCP‐1* expression

**Figure 4:**
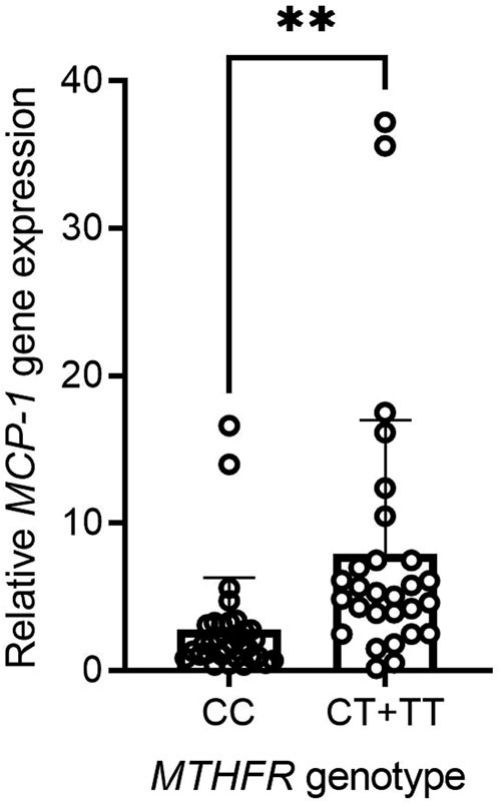
Influence of *MTHFR* rs1801133 polymorphism on *MCP‐1* gene expression in patients with AA. Expression levels are presented as mean ± *SD*. Asterisk indicates a significant *p*‐value (*p* < .05). AA, alopecia areata; MCP‐1, monocyte chemoattractant protein‐1; MTHFR, methylene‐tetrahydrofolate reductase; *SD*, standard deviation

**Figure 5:**
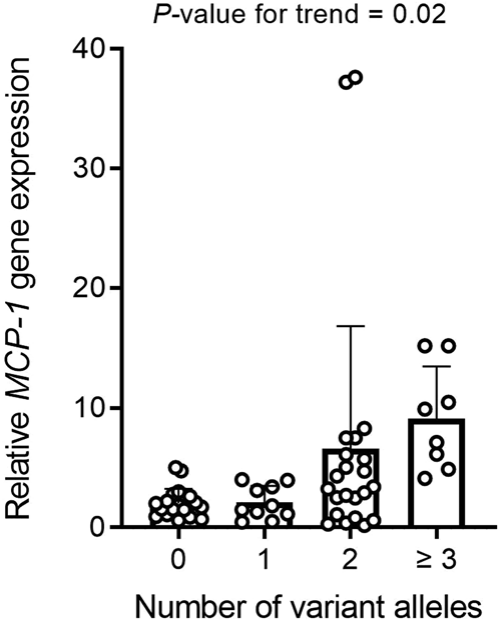
*MCP‐1* gene expression levels in AA patients, stratified according to the number of allelic variants (*MCP‐1* rs1024611 and *MTHFR* rs1801133). Expression levels are presented as mean ± *SD*. AA, alopecia areata; MCP‐1, monocyte chemoattractant protein‐1; MTHFR, methylene‐tetrahydrofolate reductase; *SD*, standard deviation

To test a possible influence of *MTHFR* variation on *MCP‐1* expression in AA, an analysis was conducted to compare the levels of *MCP‐1* gene expression in patients with wild‐ or polymorphic genotypes. As shown in Figure 4, our data indicated that patients with *MTHFR* T‐allele (CT + TT) had a significantly higher *MCP‐1* expression (*p* = .003). Since carrying *MTHFR* T‐allele and *MCP‐1* G‐allele are associated with the higher expression of *MCP‐1*, a test was applied to uncover the effect of the simultaneous presence of minor alleles of both variations on the *MCP‐1* gene expression. To this end, patients were stratified into four subgroups, including zero to three or more polymorphic alleles (*MCP‐1* rs1024611 or *MTHFR* rs1801133), and an analysis was applied to evaluate the levels of *MCP‐1* gene expression (Figure 5). Relative *MCP‐1* gene levels rose from 1.9 ± 0.26 in patients with no polymorphic variants to 9.4 ± 1.6 in patients who had three or more variant alleles at both polymorphic loci. Elevation of *MCP‐1* expression levels was significantly associated with the total number of variant alleles (*p* = .02).

### 3.5 *MCP‐1* and *MTHFR* polymorphisms are not associated with AA

In this study, genotype and allele distributions of the *MCP‐1* (rs1024611) and *MTHFR* (rs1801133) gene polymorphisms were investigated in patients with AA and the control subjects which is shown in Table 1. There was no statistically significant difference in terms of the genotypic distribution of *MCP‐1* and *MTHFR* variations between AA patients and controls (*p* = .78 and *p* = .39, respectively). Similarly, analysis of *MCP‐1* and *MTHFR* alleles demonstrated that the variant allele frequencies were similar in AA patients and controls (*p* = .79 and *p* = .37, respectively). Logistic regression analysis was also shown that there is no association between the gene polymorphisms of *MCP‐1* or *MTHFR* and the baseline clinical and demographical characteristics of the AA patients, such as age, age of onset, gender, anemia, family history, eczema, and nail dystrophy (Table 2).

**Table 1:** Genotype and allele frequencies of *MCP‐1* rs1024611 and *MTHFR* rs1801133 gene variations

Gene polymorphism	Genotype/allele	Patient (%)	Control (%)	OR	95% CI	*p*‐value
*MCP‐1* rs1024611	AA	65	33.3	1.07	0.63–1.83	.78
	AG	36.6	60			
	GG	20	6.6			
	A	61.6	63.3	1.07	0.36–1.81	.79
	G	38.3	36.6			
*MTHFR* rs1801133	CC	53.3	60	1.27	0.72–2.22	.39
	CT	36.6	33.3			
	TT	10	6.6			
	C	71.6	76.6	1.29	0.72–2.32	.37
	T	28.3	23.3			

*Note*: Logistic regression analysis was applied for assessment of genotype distribution between patients and controls. A *p*‐value of less than .05 was considered statistically significant.

Abbreviations: CI, confidence interval; MCP‐1, monocyte chemoattractant protein‐1; MTHFR, methylenetetrahydrofolate reductase; OR, odds ratio.

**Table 2:** Association between polymorphic and wild genotypes of *MCP‐1* rs1024611 and *MTHFR* rs1801133 variations and clinical information in patients with AA

	*MCP‐1* rs1024611					*MTHFR* rs1801133				
Characteristics	**AA** = **26**	**AG** + **GG** = **34**	**OR**	**95% CI**	***p*** **‐value**	**CC** = **32**	**CT** + **TT** = **28**	**OR**	**95% CI**	***p*** **‐value**
Age (years)	30.60 ± 2.21	26.64 ± 1.83	0.96	0.92–1.01	.15	28.78 ± 2.18	28.08 ± 1.86	0.99	0.95‐ 1.04	.80
Age of onset (years)	21.43 ± 2.62	21.14 ± 2.26	0.99	0.96–1.03	.93	21.96 ± 2.45	20.44 ± 2.35	0.99	0.95‐ 1.03	.65
Gender (male/female)	14/12	19/15	0.92	0.33–2.57	.87	16/16	17/11	0.64	0.23‐ 1.80	.40
Anemia	7	8	1.19	0.37–1.87	.76	9	6	1.45	0.43‐ 4.72	.55
Family history	4	8	0.59	0.15–2.29	.43	6	6	0.84	0.23‐ 3.00	.79
Eczema	3	5	0.75	0.16–3.50	.72	4	4	0.85	0.19‐ 3.80	.83
Nail dystrophy	8	9	1.25	0.39–3.81	.71	9	8	0.97	0.31‐ 3.01	.96

Note: Logistic regression analysis was applied for the assessment of wild – and polymorphic genotypes in patients. A *p*‐value of less than .05 was considered statistically significant.

Abbreviations: CI, confidence interval; MCP‐1, monocyte chemoattractant protein‐1; MTHFR, methylenetetrahydrofolate reductase; OR, odds ratio.

## 4 DISCUSSION

The current study investigated the *MCP‐1* and *MTHFR* gene polymorphisms in Iranian patients with AA to uncover a possible association between these variations and the progression of AA. The results showed no significant difference in the distribution of *MCP‐1* rs1024611 and *MTHFR* rs1801133 genotype frequencies between the AA patients and the healthy controls. However, elevated levels of *MCP‐1* gene expression were noticed in the patients carrying minor T‐allele of *MTHFR* rs1801133, which could have a potential effect on the AA phenotype.

AA is a T cell‐mediated autoimmune disease, characterized by infiltrating T cells surrounding the hair follicle.^26^ MCP‐1 is expressed by various cell types such as T cells, which plays a significant role in attracting and orchestrating the migration of inflammatory cells in pathological conditions, such as autoimmune diseases.^10^ Despite being a key chemokine involved in initiating autoinflammatory tissue damage,^27^ the aberrant expression of the *MCP‐1* or a possible impact of polymorphisms in the regulatory regions of the gene on susceptibility in AA has not been well investigated. It has been shown that high expression levels of TH1–related chemokines such as MCP‐1 affect the attraction of monocytes around the hair bulb.^1^ Similarly, MCP‐1 is significantly increased in sera from patients with AA compared with the healthy controls.^28^ Besides, variations in the *MCP‐1* gene such as a biallelic A/G polymorphism at the promoter region (rs1024611) affect the transcriptional activity of the *MCP‐1* gene.^29^, ^30^ There are controversial data regarding the association between *MCP‐1* gene polymorphism and autoimmune diseases. While some studies have shown an association between this variation and susceptibility to rheumatoid arthritis or Crohn's disease, it failed to be significant in other autoimmune diseases.^18^ Also, the meta‐analyses suggested that the *MCP‐1* rs1024611 polymorphism may confer susceptibility to Asian patients with rheumatoid arthritis and European patients with Crohn's disease, indicating the involvement of ethnicity in susceptibility to disease development.^18^ In the present study, we demonstrate no association between *MCP‐1* rs1024611 variation and AA. These data are in agreement with a single investigation performed by Hong et al.^20^ in the Korean population with no genetic association between the rs1024611 polymorphism of the *MCP‐1* gene and susceptibility to AA.

There is a body of evidence indicating a link between folate concentration and altered expression of MCP‐1, where aberrant folate/homocysteine metabolism, as well as altered MCP‐1 concentrations, are considered as the two potential pathologic factors in various autoinflammatory diseases.^12^, ^13^, ^31^ MTHFR, a key enzyme converting 5,10‐methylenetetrahydrofolate into 5‐methylenetetrahydrofolate, provides the methyl group in the converting process of homocysteine to methionine. Genetic defects of the MTHFR enzyme transcription is one of the major cause of hyperhomocysteinemia.^32^ Herein, the common variation rs1801133 in the *MTHFR* gene is involved in the alteration of MTHFR enzyme activity, which is involved in susceptibility to various diseases, such as autoimmune hair loss.^21^, ^33^ Besides, the TT genotype is responsible for the reduced activity of the MTHFR enzyme and as a consequence, increased concentrations of homocysteine.^34^ Activation of the NF‐κB transcription factor by hyperhomocysteinemia could in turn increases the expression of inflammatory cytokines and chemokines, such as MCP‐1.^11^ There are contradictory results regarding an association between *MTHFR* rs1801133 variation and susceptibility to the diseases. While some reports indicating an effect of this variation on the risk of AA or multiple sclerosis diseases in the Turkish population,^21^, ^35^ Mao et al.^36^ showed that T‐allele of *MTHFR* rs1801133 is associated with a reduction in the risk of Graves' disease, indicating a significant protective effect. Other studies failed to confirm these results in patients with atherosclerosis disease.^37^, ^38^ In our study, no association was found between *MTHFR* rs1801133 and susceptibility to AA, which is similar to the later studies. However, our data demonstrated a higher *MCP‐1* expression in the patient with AA carrying *MTHFR* T‐allele, indicating a possible effect of *MTHFR* variation on MCP‐1 production in inflammatory conditions. In line with these results, Hammons et al. observed significantly higher MCP‐1 concentrations in T carriers of *MTHFR* rs1801133 than CC homozygotes.^31^ These findings may appear to be mediated through an inflammatory stimulus‐driven, which could induce *MCP‐1* transcription activity via an NF‐κB‐dependent mechanism.

Having investigated the influence of *MCP‐1* and *MTHFR* variations in altered levels of *MCP‐1* gene expression, a hypothesis was generated that interaction between both genetic polymorphisms may have a synergistic effect on the *MCP‐1* gene expression. This was confirmed by the findings for *MCP‐1* levels, showing nearly five times higher levels in the carrier patients with three or four variants of *MCP‐1* rs1024611 and *MTHFR* rs1801133 compared with noncarriers. Nevertheless, despite an association was illustrated with *MCP‐1* levels, susceptibility to AA is not influenced by the interaction of the genotypes, as a similar distribution of these allelic variants was noticed in the patient and control subjects. Therefore, since the polymorphic genotypes of *MCP‐1* and *MTHFR* can potentially determine the inflammatory status, it might contribute to the pathophysiology of AA.

In summary, our findings indicate that while *MCP‐1* rs1024611 and *MTHFR* rs1801133 variations are not associated with AA risk, they could be involved in the disease pathogenesis by alteration of *MCP‐1* activity. Despite the limitations of the present study such as a small sample size, which may result in missing significant associations, our observations indicated that *MCP‐1* rs1024611 and *MTHFR* rs1801133 variations are associated with inflammatory *MCP‐1* activity that could be helpful for further investigations with a larger sample size.

## CONFLICT OF INTERESTS

The authors declare that there are no conflict of interests.

## AUTHOR CONTRIBUTIONS

Pardis‐Sadat Tabatabaei‐Panah, Mahsa Hajihasani, and Mahsa Mousavi participated in the experiments. Hamideh Moravvej provided the samples and performed the diagnosis of the disease. Pardis‐Sadat Tabatabaei‐Panah and Reza Akbarzadeh designed the study and performed data analysis. Reza Akbarzadeh wrote the manuscript and Ralf J. Ludwig critically revised the manuscript.

## Data Availability

All data sets generated for this study are included in the manuscript.
